# Real-World Results of Ocrelizumab Treatment for Primary Progressive Multiple Sclerosis

**DOI:** 10.1155/2020/5463451

**Published:** 2020-06-15

**Authors:** K. Daniels, P. B. van der Nat, S. T. F. M. Frequin, P. J. van der Wees, D. H. Biesma, E. L. J. Hoogervorst, E. M. W. van de Garde

**Affiliations:** ^1^Department of Value-Based Healthcare, St. Antonius Hospital, Utrecht/Nieuwegein, Netherlands; ^2^Radboud University Medical Center, Radboud Institute for Health Sciences, Scientific Center for Quality of Healthcare (IQ healthcare), Netherlands; ^3^Department of Neurology, St. Antonius Hospital, Utrecht/Nieuwegein, Netherlands; ^4^Department of Internal Medicine, University Medical Centre Utrecht, Utrecht, Netherlands; ^5^Department of Clinical Pharmacy, St. Antonius Hospital, Utrecht/Nieuwegein, Netherlands; ^6^Division of Pharmacoepidemiology and Clinical Pharmacology, Faculty of Science, Utrecht University, Utrecht, Netherlands

## Abstract

**Background:**

Recently, ocrelizumab (Ocrevus®) was approved for the treatment of primary progressive multiple sclerosis (PPMS) based on data from the ORATORIO clinical trial. Real-world data about the clinical effectiveness of ocrelizumab has yet to be gathered.

**Objective:**

The aim of this study was to provide data about the clinical effectiveness of ocrelizumab for patients diagnosed with PPMS in a real-world setting.

**Methods:**

We conducted a retrospective cohort study of all patients with PPMS who started ocrelizumab treatment (*n* = 21) in St. Antonius Hospital (Utrecht/Nieuwegein, the Netherlands) between April 2018 and December 31, 2018. Primary outcome was pre- versus post-ocrelizumab disability worsening rate (from 96 weeks prior to first ocrelizumab administration up to 24 weeks post first ocrelizumab administration).

**Results:**

Disability worsening rate while on treatment significantly differed (lower) from disability worsening rate in pre-treatment period (*Z* = −2.81, *p* ≤ .01). Three out of 17 patients showed a clinically relevant improvement in disability status after treatment start.

**Conclusion:**

Ocrelizumab can stabilize disability progression in patients with PPMS. Some patients even showed a clinically relevant improvement in disability status. Further research should help to identify which patients benefit most from ocrelizumab.

## 1. Introduction

Traditionally, outcomes of drug treatments are mostly assessed in randomized controlled trials (RCTs). However, very often, the FDA/EMA-approved indication for the administration of a drug treatment in clinical practice is eventually broader than the inclusion criteria of the RCT. Also, even within the inclusion criteria of the original RCT, the real-world patient population could be more heterogeneous than the patient population in a RCT [1, 2]. Because of variances in age, disease activity, or comorbidity [3–5], a drug can be more effective, less effective, or even not effective in real-world clinical practice.

In the field of multiple sclerosis (MS), there is a growing interest in the use of real-world data to evaluate outcomes of drug treatments [6–11]. Some disease-modifying therapies (DMTs) for MS have proven their efficacy in randomized clinical trial (RCT) but were not always equally effective for patients in real-world clinical practice [6, 7]. Recently, the first FDA- and EMA-approved drug, ocrelizumab (Ocrevus®), for the treatment of primary progressive MS (PPMS) was introduced. The ORATORIO clinical trial demonstrated the efficacy of ocrelizumab over placebo in a selected group of patients diagnosed with PPMS [12–14]. Primary efficacy endpoint was 12-week confirmed disability progression after treatment initiation, with percentages of 32.9% versus 39.3% for ocrelizumab and placebo, respectively [12]. To the best of our knowledge, no data is available yet about the effectiveness of ocrelizumab in PPMS in regular practice. The aim of this study was to provide data about the clinical effectiveness of ocrelizumab for patients diagnosed with PPMS in a real-world setting.

## 2. Method

### 2.1. Design and Setting

This study is a retrospective cohort study conducted at St. Antonius Hospital (Utrecht/Nieuwegein, the Netherlands), a large nonacademic hospital in the center of the Netherlands. At St. Antonius Hospital, ocrelizumab was available to patients with PPMS since Dutch market access in April 2018.

### 2.2. Patients

We studied all patients, diagnosed with PPMS according to the most recent McDonald criteria [15], who started ocrelizumab treatment in the period from Dutch market access (April 2018) until the end of 2018. As this study was a “real-world study,” no inclusion or exclusion criteria were applied beforehand. Baseline characteristics that were collected contained age, gender, time since MS symptoms, time since PPMS diagnosis, previously used DMTs, B-cell-targeted therapies or other immunosuppressive medications, presence of oligoclonal bands in cerebrospinal fluid or elevated IgG index, history of RRMS, SPMS, or PRMS, contraindications to MRI, and side effects from oral or intravenous glucocorticoids. Baseline was set on the date of the first ocrelizumab administration. Baseline characteristics were also used to determine whether our study population could have been eligible or not eligible for the ORATORIO clinical trial [12].

### 2.3. Procedure

As part of their clinical treatment, patients diagnosed with PPMS received two 300 mg ocrelizumab injections via an intravenous infusion at St. Antonius Hospital 14 days apart. Treatment was administered in a day-care setting overseen by a neurologist. Following regular procedures, patients were seen by a neurologist every twelve weeks, both pre-ocrelizumab administration and post-ocrelizumab administration.

### 2.4. Study Outcomes and Data Collection

The primary outcome of this study was pre- versus post-ocrelizumab disability worsening rate (from 96 weeks prior to first ocrelizumab administration up to 24 weeks post first ocrelizumab administration). Disability status was measured in Expanded Disability Status Scale (EDSS) scores [16] by neurologists certified in EDSS rating. The EDSS scale is scored in 0.5 unit increments and ranges from 0 to 10 points, with 0 indicating “totally healthy” and 10.0 indicating “death due to MS.” An increase of minimal 0.5 points on the EDSS scale was defined as disability progression. For comparison purposes to the ORATORIO study, the 12-week confirmed disability progression was collected in the pre-ocrelizumab treatment period among all patients whose EDSS scores were available from 96 weeks prior to first ocrelizumab administration up to first ocrelizumab administration (baseline) (*n* = 17). Patients of whom clinical history was not fully available due to a transfer from another hospital were not included in this comparison.

Data, i.e., treatment outcomes, were either retrieved directly from patient files or approximated from documented neurological examinations. Data was collected by KD and independently verified by SF who is certified in EDSS rating. In case of discrepant findings, consensus was reached. REDCap CRF was used to store extracted data, guaranteeing anonymity and privacy of the patient cohort during and after this study. In case of missing EDSS values, e.g., due to patients missing a 12-week outpatient visit, the mean EDSS score of the previous and next outpatient visit was manually imputed. All missing values (9.7%) occurred in pre-ocrelizumab treatment period; no values were missing in post-ocrelizumab treatment.

### 2.5. Statistical Analysis

IBM SPSS (version 24.0) was used to execute statistical analyses. Descriptive statistics were used for baseline characteristics. To assess whether the post-ocrelizumab disability worsening rate (EDSS at 24 weeks post treatment minus EDSS at treatment/number of weeks) differed from the pre-ocrelizumab disability worsening rate (EDSS at treatment minus EDSS at 96 weeks prior to treatment/number of weeks), a paired Wilcoxon Signed-Rank Test was executed. To assess whether the real-world pre-ocrelizumab population was clinically similar to the ORATORIO placebo arm, in terms of 12-week confirmed disability progression over time, a time-to-event curve was generated. External reference data regarding disability progression over time was extracted from the ORATORIO clinical trial [12] to create this graph. Theoretically, the level of disease progression in the pre-ocrelizumab period should match the level of disease progression as seen in the placebo group from ORATORIO. No sample size calculations were made beforehand because of the explorative nature of the study.

## 3. Results

### 3.1. Study Population and Cohort Characteristics

From April 2018 through the end of 2018, a total of 21 patients diagnosed with PPMS started ocrelizumab treatment. The characteristics of the patients at baseline are provided in Table 1. In short, the mean age was 52 years and patients were diagnosed on average 5 years before. The mean EDSS score at time of ocrelizumab start was 5.3 (SD = 1.1). Based on the exclusion and inclusion criteria from the ORATORIO clinical trial [12], a total of *n* = 11 patients in our study cohort could have been eligible for the ORATORIO clinical trial whereas *n* = 10 patients would not have been eligible for the ORATORIO clinical trial. The main reasons for noneligibility were higher age (*n* = 6), previously used B-cell-targeted therapies or other immunosuppressives (*n* = 1), history of RRMS, SPMS, or PRMS diagnosis (*n* = 1), and shorter history of disease (*n* = 2) (see supplemental Table 1). History of RRMS, SPMS, or PRMS diagnosis involved one patient who was previously misdiagnosed with RRMS and had previously received a DMT (Table 1). The diagnosis of this patient was altered into PPMS by the neurologist based on the patient's clinical disease course more than one year prior to ocrelizumab administration. All patients fulfilled the FDA/EMA-approved indication for ocrelizumab at time of administration, and in none of the cases, infusion-related reactions were recorded (compared to 39.9% infusion-related reactions in ORATORIO trial [12]). Seventeen patients reached the 24-week follow-up point. For other patients, treatment was administered too recently to already report on 24-week EDSS scores (*n* = 2) or there was not enough historical data available due to the patient coming to the clinic for a second opinion or referral (*n* = 4) or both (*n* = 1).

**Figure 1:**
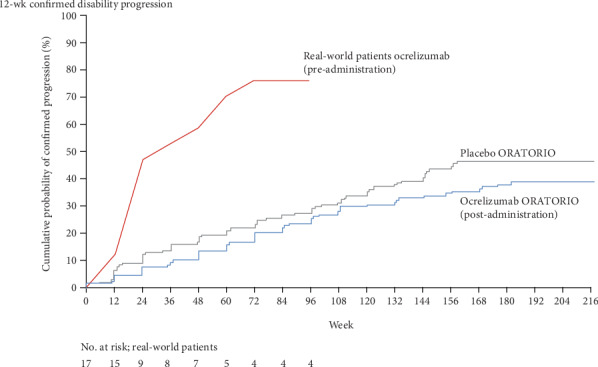
Time-to-event: 12-week confirmed disability progression of real-world pre-ocrelizumab population (*n* = 17) and the ORATORIO placebo arm. ORATORIO data were adapted from the original ORATORIO publication of Montalban et al. [12].

Figure 1 displays patients' clinical disease course in two years (96 weeks) before treatment. During this pre-treatment period, 76.5% of the real-world patient population showed 12-week confirmed disability progression compared to approximately 27% of the ORATORIO placebo group [12].

### 3.2. Effectiveness of Ocrelizumab

**Figure 2:**
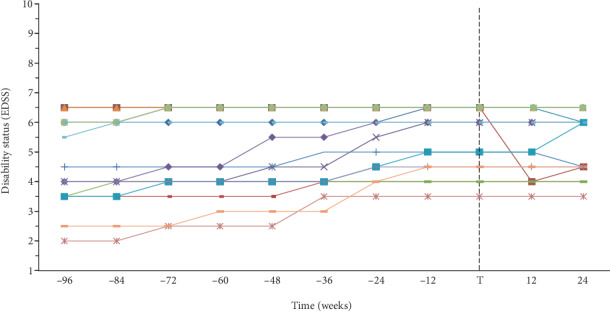
Pre- versus post-ocrelizumab disability worsening of patients diagnosed with PPMS (*N* = 17).

Primary outcome of this study was pre- versus post-ocrelizumab disability worsening rate (Figure 2). Disability worsening rate in pre-treatment period significantly differed from disability worsening rate while on treatment (*Z* = −2.81, *p* ≤ .01), indicating that the disability progression rate per 12 weeks was lower after the first ocrelizumab administration (*M* = −.06, SD = .29) than before treatment (*M* = .09, SD = .09). This suggests that ocrelizumab stabilized the disability progression. Furthermore, three out of 17 patients indicated that their disability status improved over time after treatment administration. One patient who gained health by 2.5 EDSS points had improved in walking ability (walker vs. ambulatory without aid), walking distance (≥20 meters with aid vs. ≥500 meters without aid), and balance. The other two patients who gained health by 0.5 EDSS points also reported an increase in walking ability (constant bilateral assistance vs. unilateral assistance) or walking distance (≥200 meters vs. ≥300 meters).

## 4. Discussion

This study provides first insight in the effect of ocrelizumab for the treatment of PPMS in a real-world clinical setting. This real-world data study confirmed that ocrelizumab can stabilize disability progression in patients with PPMS. Interestingly, three out of 17 patients even showed clinically relevant improvement in disability status. Ocrelizumab appears to be effective even though this real-world study population seems to have a more rapidly worsening form of PPMS than the study population in the ORATORIO clinical trial.

To the best of our knowledge, this study is the first to provide data about the real-world clinical effectiveness of ocrelizumab in patients with PPMS. This study used an unselected cohort, consisting of a heterogeneous group of patients that were all treated according to the FDA/EMA-classified indication for ocrelizumab. Additionally, it is interesting to see that only half of our real-world population could have been eligible for the ORATORIO clinical trial, meaning that the effect of ocrelizumab on this variety of patients has never been evaluated before. Further research is recommended to investigate whether the effect of ocrelizumab is different for ORATORIO eligible and not eligible patients. Unique in this study is the presentation of the absolute values of disability status (EDSS) over time, not just the cumulative probability of 12-week confirmed disability progression as was provided in the ORATORIO clinical trial. The absolute values of disability status provide additional insight in the severity of disability progression of every single patient. For example, a patient that starts with an EDSS score of 4.5 and reaches an EDSS score of 6.5 after two years has a different disability progression curve than a patient that starts with an EDSS score of 4.5 and reaches an EDSS score of 5.5 after two years. This insight is relevant to patients and can only be gained by presenting absolute values as was done in this study.

Although this study succeeded at reaching its aim, there were some limitations that should be considered when discussing this study's findings. First, one of the major limitations is the small sample size of this study due to the recent market introduction of ocrelizumab and the relatively low prevalence of PPMS. Therefore, our sample had insufficient power to perform subgroup analyses or to statistically compare our sample with that of the ORATORIO clinical trial. Second, due to the recent market introduction of ocrelizumab, the follow-up period of this study is relatively short (24 weeks). On the other hand, the ORATORIO clinical trial showed that the stabilizing effect of ocrelizumab on disability progression was primarily seen in the first 12 weeks [12]. After 12 weeks, the placebo group and the ocrelizumab group seemed to continue in approximately the same progression rate. This suggests that ocrelizumab does not slow progression in every patient but only in a subgroup. Possibly, these are our three out of 17 patients who experienced an improvement in disability status in the weeks following ocrelizumab administration. Unfortunately, the ORATORIO study report [12] does not provide data about how many patients' disability status improved and whether this happening was predictive for the 12-week confirmed disability progression outcome. Due to our short follow-up time, we could not explore this further at this moment. Other limitations of this study are standard for a retrospective study, being missing data, interpretation errors in medical records, and the absence of a control group, making this study a level IV evidence study [17].

In conclusion, this study showed that ocrelizumab can stabilize disability progression in patients with PPMS, even though this real-world study population seems to have a more rapidly worsening form of PPMS than the study population in the ORATORIO clinical trial. Our finding of some patients showing clinically relevant improvement in disability status is an important lead for further research into which patients benefit most from ocrelizumab.

## Figures and Tables

**Table 1:** Baseline characteristics of real-world patient population (*n* = 21), displayed separately for patients who met the eligibility criteria for the ORATORIO clinical trial and patients who did not meet the eligibility criteria.

	Real world			ORATORIO^a^
	ORATORIO not eligible (*N* = 10)	ORATORIO eligible (*N* = 11)	Total (*N* = 21)	Ocrelizumab (*N* = 488)
Age (yr)				
Mean (SD)	54.90 (8.03)	49.55 (3.04)	52.09 (6.42)	44.7 (7.9)
Median (range)	56.50 (37-67)	49.00 (46-54)	53.00 (37-67)	46.0 (20-56)
Female sex—no. (%)	5 (50.0)	9 (81.8)	14 (66.7)	237 (48.6)
Time since onset of MS symptoms (yr)				
Mean (SD)	6.76 (4.16)	7.42 (4.70)	7.11 (4.35)	6.7 (4.0)
Median (range)	6.04 (.63-12.47)	5.66 (1.95-14.53)	5.95 (.63-14.53)	6.0 (1.1-32.9)
Time since diagnosis of PPMS (yr)				
Mean (SD)	4.45 (4.42)	5.32 (4.39)	4.9 (4.31)	2.9 (3.2)
Median (range)	3.30 (.23-12.47)	3.75 (.21-14.53)	3.75 (.21-14.53)	1.6 (.1-16.8)
No previous use of disease-modifying therapy—no. (%)	9 (90.0)	11 (100)	20 (95.2)	433 (88.7)
If yes: dimethyl fumarate	1 (10.0)	0 (0)	1 (4.8)	NA
Score on EDSS				
Mean (SD)	4.9 (.99)	5.72 (1.15)	5.33 (1.13)	4.7 (1.2)
Median (range)	4.75 (3.5-6.5)	6.50 (3.5-6.5)	6.0 (3.5-6.5)	4.5 (2.5-7.0)

^a^ORATORIO data were adapted from Montalban et al. [12].

## Data Availability

Raw data were generated at St. Antonius Hospital. Derived data supporting the findings of this study are available from the corresponding author KD on request.

## Supplementary Materials

Supplementary Materials Supplemental Table 1: eligibility of real-world population in St. Antonius Hospital (*n* = 21) to ORATORIO clinical trial, separated for each eligibility criteria.
