# Supplementary material for: Real-World Results of Ocrelizumab Treatment for Primary Progressive Multiple Sclerosis
Source: Mult Scler Int. 2020 Jun 15;2020:5463451. doi: 10.1155/2020/5463451 (PMC7313157; doi:10.1155/2020/5463451)
Supplement: Supplementary Materials — Supplemental Table 1: eligibility of real-world population in St. Antonius Hospital (n = 21) to ORATORIO clinical trial, separated for each eligibility criteria. [file 5463451.f1.pdf]

Supplemental table 1. Eligibility of real-world population in St. Antonius Hospital (n = 21) to ORATORIO clinical trial; separated for each eligibility criteria[a]

	Number of patients eligible (%)
Age 18-55 years	15 (71.4)
EDSS score of 3.0 to 6.5 at baseline	21 (100)
Pyramidal functions component of the functional systems Scale of ≥ 2	21 (100)
Time since first MS symptoms ≤ 15 years (EDSS > 5.0 at baseline)	21 (100)
Time since first MS symptoms ≤ 10 years (EDSS ≤ 5.0 at baseline)	19 (90.5)
History of elevated IgG index or ≥ 1 IgG oligoconal band in cerebrospinal fluid.	21 (100)
No History of RRMS, SPMS or PRMS	20 (95.2)
No contraindication to MRI	21 (100)
No side effects from oral or intravenous glucocorticoids	21 (100)
No previous treatment with B-cell-targeted therapies or other immunosuppressive medications	20 (95.2)

[a]ORATORIO eligibility criteria consisted of seven inclusion criteria and four exclusion criteria[12]
